# DFENet: A diverse feature extraction neural network for improving automatic modulation classification accuracy in wireless communication systems

**DOI:** 10.1371/journal.pone.0341020

**Published:** 2026-01-21

**Authors:** Ha-Khanh Le, Van-Phuc Hoang, Van-Sang Doan

**Affiliations:** 1 Institute of System Integration, Le Quy Don Technical University, Hanoi, Vietnam; 2 Faculty of Communication and Radar, Vietnam Naval Academy, Nha Trang, Vietnam; Beijing Institute of Technology, CHINA

## Abstract

In this paper, a convolutional neural network (CNN) model, named DFENet, is composed of multi-branch blocks for diverse feature extraction (DFE) to improve the accuracy of automatic modulation classification (AMC) in wireless communication systems. The DFE blocks primarily involve advanced processing sub-blocks designed to extract signal features from IQ (In-phase and Quadrature-phase) data using filters at multiple scales. By extracting diverse intrinsic features, the convolutional layers with different filter sizes can prevent overfitting and gradient vanishing problems, thus enhancing the AMC accuracy while maintaining reasonable computational complexity. Experimental results with the HisarMod2019 dataset demonstrate that the DFENet model with 4 DFE blocks and 128 filters in the convolutional layers achieved an average AMC accuracy of 82.76%, excelling particularly at low SNR levels, with an accuracy exceeding 60% at SNR = –20 dB and greater than 90% at SNR = –2 dB. For the RadioML2018.01A dataset, the proposed model yielded an accuracy of AMC greater than 93% for SNR >6 dB. In comparison, our model outperforms other state-of-the-art models in terms of accuracy while maintaining reasonable computational complexity and fast execution time.

## 1 Introduction

Nowadays, automatic modulation classification (AMC) plays a crucial role in modern radio frequency (RF) systems, including MIMO communications, cognitive radio, RF based drone surveillance and electronic intelligence. In a typical wireless communication system, transmitters and receivers exchange waveforms following a predefined protocol. However, transmitted signals are often distorted by channel impairments such as noise, multipath fading, and frequency offset. Hardware and oscillator design limitations further complicate the distinction between modulation schemes, particularly under multipath fading conditions [1,2]. As a critical intermediate step between signal detection and demodulation, AMC identifies the modulation scheme to enable proper signal processing. With the growing complexity of wireless technologies, modulation schemes are becoming more diverse to address advanced communication challenges [3]. This demands robust and efficient AMC models capable of operating in harsh RF environments [4,5]. Recent advances in deep learning (DL), driven by improved algorithms, computational power, and dataset availability, have significantly transformed fields like computer vision, speech recognition, and natural language processing. For AMC, high-quality datasets are essential, as they form the foundation for training high-performance deep learning models.

Deep learning has become a pivotal technique in AMC due to its ability to autonomously extract intricate features from raw signal data [6]. Among various DL architectures, convolutional neural networks (CNNs), particularly ResNet [7], have emerged as leading solutions, demonstrating remarkable success in AMC tasks [8,9]. A key advantage of CNNs lies in their ability to process IQ signals by reshaping them into dimensional structures similar to images, leveraging their proven strength in visual pattern recognition. This approach has enabled CNN-based models to surpass traditional feature-based methods in modulation classification accuracy. Various CNN-based approaches have been deployed that include modifications to standard architectures, simplified network designs, and advanced models incorporating state-of-the-art techniques. For example, O’Shea et al. [10] modified VGG and ResNet architectures by introducing 1D convolutional layers, optimizing them for radio signal classification. In contrast, the CNN architectures proposed in [11–13] employed shallow networks with only a few convolutional layers directly linked to activation layers. This constrained design limited their learning capacity, resulting in suboptimal classification accuracy. To improve AMC performance, Huynh-The et al. [14] developed MCNet, an AMC-optimized architecture that balances learning efficiency with computational demands through multiple 1D convolutional feature learning blocks. In another study, a Long Short-Term Memory (LSTM) network was applied to classify various modulated signals [15]. In [16], a novel DL architecture for AMC was proposed by merging VGG and ResNet. In addition, a three-stream multimodal fusion approach was introduced in [17,18], where a multimodal structure was constructed using CNN and LSTM based on modulated IQ symbols from signals. Beyond direct utilization of original signals, an alternative form of AMC methods has been proposed in [19,20] by transforming the original input signals into matrices and then integrating them with convolutional networks for AMC. These advances validate the robust performance of deep learning in diverse signal classification scenarios. Recent studies have also addressed the broader context of multi-node and multi-type signal detection. For example, Huijun Xing et al. in [21] introduces a framework that jointly performs signal detection and AMC in co-existing multi-signal environments. Similarly, Jagannath et al. [22] leverage a multi-task learning paradigm to simultaneously detect and classify diverse modulation types in complex wireless environments. These works illustrate promising directions for combining detection and classification in complex wireless settings.

Current AMC models often fail to explicitly address the distinct challenges posed by low- and high-SNR conditions, resulting in suboptimal performance across diverse noise environments [23–25]. At low SNRs, the signal amplitude frequently approaches or falls below the noise floor, causing severe overlap between signal and noise components and leading to ambiguous or distorted modulation patterns [26,27]. Conversely, high-SNR signals retain clear temporal and spectral structures with minimal noise contamination, enabling easier feature extraction and classification. These contrasting characteristics highlight the need for a unified architecture capable of capturing both fine-grained features under heavy noise and global patterns in cleaner conditions. This motivates our design of DFENet, where multi-scale feature extraction and feature enhancement blocks jointly ensure robust representation learning across the full SNR spectrum, addressing limitations in prior approaches [28]. Besides, by explicitly incorporating multi-scale convolutional branches and normalization mechanisms, DFENet directly links the observed challenges of low- and high-SNR signals with concrete architectural innovations. As demonstrated in the experimental sections, this design leads to substantial gains in classification accuracy across a wide SNR range, thereby validating the effectiveness of the proposed approach.

Furthermore, with the rapid growth of the Internet of Things (IoT), the deployment of AMC models on resource-constrained devices has become increasingly critical. These devices, which operate in complex wireless environments such as industrial sensor networks, face challenges such as high traffic volumes, wide spectrum utilization, and stringent requirements for accurate signal identification. While many deep learning-based AMC methods have been developed, a significant number still rely on image feature extraction or complex signal conversion processes, which result in high computational overhead during both training and testing phases [29]. These inefficiencies pose substantial obstacles, particularly in time-sensitive tasks, highlighting the challenge of developing an AMC approach that effectively balances high accuracy with low computational demands.

In this study, we introduce a high-performance CNN architecture, named DFENet, that is designed for AMC under challenging channel conditions. The main contributions of our approach can be summarized as follows:

1. Advanced diverse feature extraction blocks: The DFENet architecture is centered around several advanced feature learning blocks called DFE blocks. Each block consists of multiple parallel 2-D convolutional layers that extract diverse features from the input data. To further refine these features, we employ a structure of parallel layers with different filters. This configuration improves feature extraction and enhances the model’s classification capabilities by providing a more detailed representation of the signal features. The ablation study demonstrates that removing the multi-scale structure reduces accuracy on the HisarMod2019 dataset from 80.8% to 70.59%, highlighting the essential role of diverse feature extraction.
2. Improved generalization ability: The use of multiple CNN blocks with batch normalization (BN) helps reduce the risk of over-fitting and improves accuracy by capturing details that simpler models might overlook. Experiments confirm that excluding the FEN block results in a 3.5% drop in accuracy at SNR≤0 dB, showing its importance for robust performance in adverse conditions.
3. Enhanced performance and efficiency: The simulation results of DFENet on the HisarMod 2019 and RadioML 2018.01A datasets demonstrate its effectiveness in handling multi-layer modulations. On the HisarMod2019 dataset, the network achieves an average accuracy of 82.76%, maintaining over 60% accuracy at SNR = –20 dB and exceeding 90% at SNR = –2 dB. On the RadioML2018.01A dataset, DFENet attains greater than 93% accuracy for SNR >6 dB, outperforming ResNet and MCNet by 3–5%. In addition, DFENet contains only 2.2M parameters and delivers 40% faster inference compared to ResNet, thereby achieving a favorable balance between accuracy and computational cost. These results confirm the practical applicability of DFENet to real-time AMC tasks on resource-constrained platforms.

The rest of this paper is structured as follows. Sect 2 presents the modulation recognition problem. Sect 3 details the DFENet model utilized for AMC. Sect 4 outlines the experimental setup. Sect 5 presents and analyzes the classification results, highlighting the benefits of the proposed model. Finally, Sect 6 provides a summary and concludes the study.

## 2 Radio signal modulation

**Fig 1:**
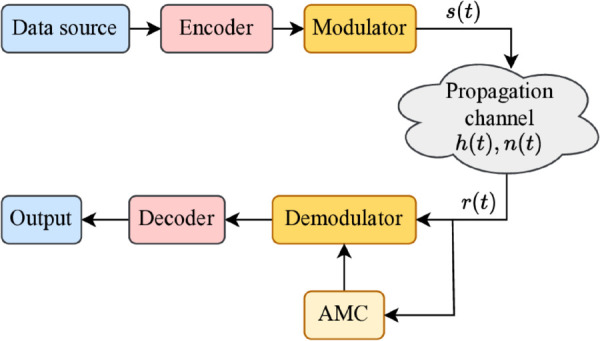
Wireless communication network.

Modulation is fundamental to wireless communication, enabling broader signal bandwidth and improved resistance to fading and interference. By converting baseband signals into high-frequency carriers, it ensures accurate, low-noise data transmission over long distances. In a typical communication system, the modulation recognition module resides at the receiver, as shown in Fig 1. Here, the AMC module plays a pivotal role by autonomously identifying the modulation scheme, a critical step for correct demodulation and data decoding. AMC enhances system flexibility by allowing receivers to adapt to varying modulation schemes and channel conditions without prior knowledge of transmitter settings. This capability is essential for adaptive modulation, optimizing spectral efficiency and signal performance in dynamic environments like 5G and LTE networks. Positioned between signal reception and demodulation, AMC processes IQ samples, classifies the modulation scheme, and ensures reliable data extraction. The performance of AMC algorithms heavily depends on accurate signal modeling and preprocessing.

In wireless communication systems, the received signal *r*(*t*) can be expressed as follows:

r(t)=s(t)*h(t)+n(t) (1)

where *s*(*t*) is the transmit signal, *h*(*t*) is the channel response and n(t) represents additive noise. In complex form, the received signal is presented as

$$r(t)=R(t)\cdot \exp(2\pi f_{c}t+\phi (t))$$ (2)

where *R*(*t*) is the envelope of the amplitude, *f*_*c*_ is the carrier frequency, and ϕ(t) is the phase of the signal. The complex signal can be decomposed into its real and imaginary parts as follows:

- Real (Re) or I component:

$$\text{Re}(r(t))=R(t)\cdot \cos(2\pi f_{c}t+\phi (t))$$ (3)

- Imaginary (Im) or Q component:

$$\text{Im}(r(t))=R(t)\cdot \sin(2\pi f_{c}t+\phi (t))$$ (4)

Consequently, the signal can be represented in complex form through its in-phase (I) and quadrature (Q) components.

## 3 DFENet model for AMC

This section presents a comprehensive description of DFENet, a novel architecture designed to enhance automatic feature learning during training while optimizing modulation classification performance during inference.

**Fig 2:**
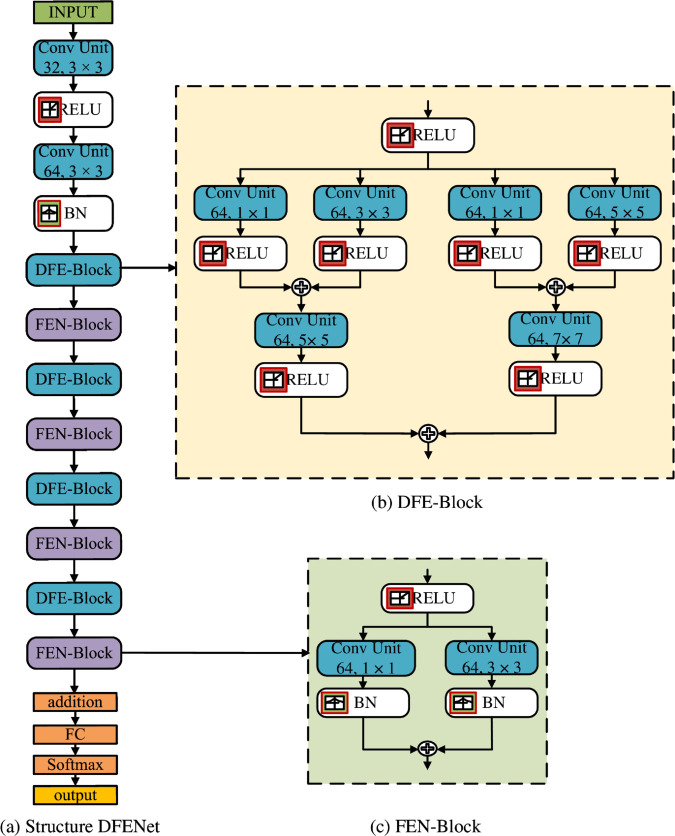
Deep CNN architecture for AMC: (a) the overall architecture of DFENet, (b) the structure of DFE Block, (c) the structure of FEN block.

DFENet employs an innovative wide-modeling framework that incorporates parallel branches with multiscale convolutional filters. This design significantly strengthens the feature extraction capability of the network by capturing diverse signal characteristics. The model processes input signals with a fixed length of 1024 samples, matching the sample intervals used in both the HisarMod2019 and RadioML2018 datasets. As illustrated in Fig 2(a), the network architecture incorporates two key processing blocks: Dynamic Feature Extraction (DFE) blocks, and Feature Extraction and Normalization (FEN) blocks. These consecutive connected blocks form the backbone of DFENet, working together to extract and refine discriminative signal features across various SNR conditions. This dual-block design enhances both high-level and fine-grained feature representation, significantly increasing the model performance in complex modulation classification tasks.

### 3.1 DFE block for diverse feature extraction

The DFE block plays a crucial role in multichannel feature extraction, utilizing filters of varying sizes to capture information from different aspects of the signal. As illustrated in Fig 2(b), this block consists of six convolutional units (Conv), which are followed by rectified linear unit (ReLU) layers. Let the input IQ signal be presented as a matrix $𝐗\in {\mathbb{R}}^{L\times 2}$, where *L* is the signal length (1024 samples), and the two columns correspond to the IQ components. The matrix **X** is processed in the convolutional layers, which applies a set of filters to extract features from the matrix **X**. The convolution operation can be expressed as follows [30]:

𝐂=Conv(𝐗)=𝐀*𝐗+𝐛 (5)

where **A** represents the convolutional filter, * denotes the convolution operation, and **b** is the bias.

Following the convolution operation, the output feature map **C** undergoes ReLU activation to introduce non-linearity into the network, enabling it to capture and learn complex patterns and relationships in the data. This step ensures that only the positive values of **C** are retained, while the negative values are set to zero. The output of the ReLU function is defined as follows:

𝐘=ReLU(𝐂)=max(0,𝐂) (6)

The six Conv units are arranged in two parallel branches. In branch 1, convolutional layers in two parallel sub-branches have filter sizes of 1×1 and 3×3, respectively. Both layers are followed by ReLU activation layers. The two sub-branches are then combined through an addition layer:

$$Y_{1}=\text{ReLU}({\text{Conv}}_{3\times 3}(Y))+\text{ReLU}({\text{Conv}}_{1\times 1}(Y))$$ (7)

Then, a convolution of the filter size 5×5 is used to refine the output:

$$Y_{1.1}=\text{ReLU}({\text{Conv}}_{5\times 5}(Y_{1.1}))$$ (8)

Branch 2 operates similarly to branch 1, but it has different filter sizes: 1×1, 5×5, and 7×7:

$$Y_{1.2}=\text{ReLU}({\text{Conv}}_{7\times 7}(\text{ReLU}({\text{Conv}}_{5\times 5}(Y))+\text{ReLU}({\text{Conv}}_{1\times 1}(Y))))$$ (9)

The two major branches are combined at the addition layer to produce the output of the first DFE block:

$$Y_{DFE}=Y_{1.1}+Y_{1.2}$$ (10)

The DFE block serves as the key component of the DFENet architecture, drawing inspiration from multiscale representation learning. Unlike conventional CNNs that utilize uniform filter sizes across layers, the DFE block incorporates parallel convolutional branches with progressively increasing filter sizes 1 × 1, 3 × 3, 5 × 5, and 7 × 7. This multi-branch design enables comprehensive feature extraction by capturing modulation characteristics across different temporal scales. Small filter sizes such as 1 × 1 and 3 × 3 effectively capture fine-grained high-frequency local features, such as instantaneous phase shifts and amplitude variations in the IQ data.

The multiscale architecture of DFE blocks employs carefully selected kernel sizes (1 × 1, 3 × 3, 5 × 5, and 7 × 7) to capture diverse temporal characteristics from raw IQ signals. Specifically, larger filters (5 × 5, 7 × 7) analyze broader temporal dependencies, enabling recognition of structural patterns in high-order modulations and long-term signal dynamics, particularly valuable in challenging scenarios with fading channels or strong noise interference. From a frequency-domain perspective, this design creates a comprehensive analysis framework: smaller kernels (1 × 1, 3 × 3) function similarly to high-pass filters, accentuating rapid local variations, while larger kernels emulate low-pass filters, extracting slowly varying features through signal smoothing. As a result, this multiscale approach provides enhanced frequency decomposition, improved feature diversity, robust generalization across modulation types, and consistent performance under varying SNR conditions.

The outputs from all parallel branches are combined through element-wise addition, enabling seamless integration of multiscale features without adding network depth or compromising inference speed. This design achieves two key advantages: (1) it enhances the richness of modulation feature representations while preserving computational efficiency, and (2) it allows flexible feature recombination at different temporal resolutions. In addition, by stacking multiple DFE blocks in sequence, the model progressively learns higher-level abstractions of modulation characteristics, forming an effective hierarchical representation. This architecture demonstrates particular strength in maintaining classification accuracy across a broad range of SNR conditions, from high-quality channels to severely degraded environments.

### 3.2 FEN block for feature extraction and normalization

Each DFE block is followed by an FEN block, which is designed to refine and normalize the features extracted by the preceding block, enhancing discrimination and reducing noise, as shown in Fig 2(c). In the FEN block, batch normalization (BN) layers are used after the convolutional units instead of ReLU layers. The operation of a BN layer is defined as follows [31]:

$${𝐘}_{\text{BN}}=\gamma \frac{𝐘-\mu_{2}}{\sqrt{\sigma^{2}+\epsilon }}+\beta$$ (11)

where, $\mu_{2}$ and $\sigma_{2}^{2}$ are the mean and variance, $\gamma_{2}$ and $\beta_{2}$ are the learnable parameters, and *ϵ* is a small value to avoid division by zero.

Regarding the block structure, the feature map ${𝐘}_{\text{DFE}}$ is passed to the FEN block, which serves to refine and stabilize the features before deeper processing. This block uses two convolutional branches with kernel sizes 1×1 and 3×3, followed by batch normalization (BN) layers instead of activation functions. Let $W_{1}^{1\times 1}$, $W_{2}^{3\times 3}$ be the respective kernels. The block output is given by:

$${𝐅}_{1}=\text{BN}({𝐘}_{\text{DFE}}*W_{1}^{1\times 1})$$ (12)

$${𝐅}_{2}=\text{BN}({𝐘}_{\text{DFE}}*W_{2}^{3\times 3})$$ (13)

$${𝐅}_{\text{FEN}}={𝐅}_{1}+{𝐅}_{2}$$ (14)

The FEN block of our model employs convolutional layers with smaller sizes of 1 × 1 and 3 × 3 to refine and optimize the features extracted by the DFE block. This process helps to suppress unnecessary noise and highlight critical features. Batch normalization is applied after each convolution layer to stabilize the training process and mitigate the vanishing gradient problem. By normalizing the features, the model learns more efficiently and converges faster. The addition operation between branches following batch normalization integrates the optimized information, producing a high-quality output that enhances the accuracy of the model in the AMC task. The FEN block ensures stabilization and optimization for modulation classification. Repetition of the DFE and FEN blocks *n* times allows the proposed model to learn deeper and extract features more effectively.

### 3.3 Fully connected layer and softmax layer

After stacking multiple DFE and FEN blocks, the output ${𝐎}_{n}$ is flatten and fed to a fully connected layer (FC), which is responsible for capturing complex non-linear relationships and extracting high-level features from the data. The operation is described as follows [32]:

$${𝐅}_{FC}={𝐖}_{FC}\cdot {𝐎}_{n}+{𝐛}_{FC}$$ (15)

where ${𝐖}_{FC}$ is the weight matrix and ${𝐛}_{FC}$ is the bias vector.

The output vector ${𝐅}_{FC}$ is then passed through a softmax layer, which converts the output into class probabilities (scores). The softmax function ensures that the sum of all predicted probabilities equals to one:

$$𝐏(i)=\frac{e^{𝐅(i)}}{\sum_{j=1}^{N}e^{𝐅(j)}}$$ (16)

where 𝐏(i) is the probability of class *i*, and *N* is the total number of classes.

### 3.4 Comparison with existing architectures

Although DFENet follows the general principle of multi-branch convolution, it differs substantially from existing models such as Inception, ResNet, and MCNet [33]. Table 1 summarizes the main architectural differences and highlights the domain-specific adaptations that make DFENet suitable for AMC tasks.

**Table 1:** Comparison of DFENet with existing multi-branch and multiscale architectures.

Model	Multi-scale Conv	Residual Connections	Feature Normalization
Inception	Yes (1×1, 3×3, 5×5)	No	BN
ResNet	No (uniform 3×3)	Yes	BN+ReLU
MCNet	Yes (multi-scale)	Partial	No FEN
**DFENet**	Yes (1×1, 3×3, 5×5, 7×7)	No deep stacking	**FEN block (BN only)**

Unlike Inception, which was designed for vision tasks and depends on pooling operations that may remove important temporal information, DFENet eliminates pooling to keep the fine time structures of IQ signals. Compared to ResNet, DFENet does not rely on stacking multiple residual blocks but instead leverages multi-scale kernels, making the model more compact while still effectively capturing both short-term symbol variations and long-term temporal dependencies. In contrast to MCNet, which also employs multi-scale convolution but lacks explicit normalization, DFENet integrates both the DFE block (diverse feature extraction) and the FEN block (feature normalization). This combination stabilizes training and enhances feature separability under noisy and low-SNR conditions. Overall, DFENet is a lightweight yet powerful model, specifically designed for automatic modulation classification (AMC) in challenging wireless environments. These differences highlight the novelty of DFENet as a domain-optimized architecture rather than a mere adaptation of existing multiscale CNNs.

Regarding the network configuration reported in Table 2, the DFENet-4block model comprises approximately 2 million parameters. This architectural design has been carefully considered to strike an optimal balance between network capacity and computational efficiency. Although this parameter count is significantly lower than that of other high-performing models, such as RepVGG (24.8 million) or Inception (21.8 million), this compact size is achieved through the efficient use of Diverse Feature Extraction (DFE) blocks. This is crucial because the core functionality of the DFE block feature pooling from parallel convolutional kernels (1 × 1,3 × 3,5 × 5,7 × 7) is essential for the model to maintain high recognition accuracy, especially in low SNR environments where rich and diverse feature extraction is necessary to mitigate the impact of noise. Therefore, the current configuration represents an optimal trade-off: minimizing computational load while retaining the deep hierarchical structure required for robust Automatic Modulation Classification (AMC).

**Table 2:** Detailed architecture of the DFENet-4block model.

Layer	Kernel Size (K)	Stride (S)	Output Shape
**Input**	**-**	**-**	** 1024×2×1 **
Conv2D/ReLu	(3×2)	1×1	1024×2×32
Conv2D/BN/ReLu	(3×2)	1×1	1024×2×64
**DFE Block**	** {1,3,5,7} **	** 1×1 **	** 1024×2×64 **
**FEN Block**	** {1,3,} **	** 2×2 **	** 512×1×64 **
**DFE Block**	** {1,3,5,7} **	** 1×1 **	** 512×2×64 **
**FEN Block**	** {1,3,} **	** 2×2 **	** 256×1×64 **
**DFE Block**	** {1,3,5,7} **	** 1×1 **	** 256×2×64 **
**FEN Block**	** {1,3,} **	** 2×2 **	** 128×1×64 **
**DFE Block**	** {1,3,5,7} **	** 1×1 **	** 128×2×64 **
**FEN Block**	** {1,3,} **	** 2×2 **	** 64×1×64 **
Fully Connected	-	-	1×1×26
Dense/Softmax	-	-	1×26

## 4 Dataset description and experiment setup

In this study, the HisarMod2019.1 [34] and RadioML 2018.01A [10] datasets are used. The HisarMod2019.1 dataset contains 26 different modulation signals belonging to five modulation groups. In this dataset, signals are affected by Gaussian noise and five types of fading channels, including ideal channel conditions, static, Rayleigh, Rician, and Nakagami-m. Each modulation type consists of 1500 signals, each with a length of 1024 IQ samples. For different SNR levels, ranging from –20 dB to 20 dB in steps of 2 dB, the dataset comprises 780,000 signals, with 520,000 used for training and the remainder for testing.

The RadioML 2018.01A dataset [10] is well-known as one of the most widely used datasets in the field of modulation classification. The modulation signals in this dataset are subjected to independent Rician multipath channels, each characterized by different delay spreads, Doppler shifts, frequency offsets, sampling time drifts caused by clock offsets, and additive white Gaussian noise, as detailed in [10]. The data set was divided into 70% for training, 15% for validation, and the remaining 15% for testing.

For the HisarMod2019.1 dataset, we trained both the proposed model and baseline models for 20 epochs using the following parameters: mini-batch size of 64, initial learning rate of 0.001 with a 10-fold reduction every 5 epochs. Regarding the RadioML2018.01A dataset, the ADAM optimizer was used for training, the maximum number of epochs is set to 90, the learning rate is initialized at 0.001, and the mini-batch size is assigned to 256. The simulations were conducted on a computer equipped with a 3.70 GHz CPU, 2x16GB RAM, and a NVIDIA GeForce RTX 3060 Ti GPU. In the next sections, the performance of the DFE model is evaluated on the HisarMod2019 and RadioML2018 datasets, and compared with contemporary networks, including ResNet, SqueezeNet, MobileNet, GoogleNet, RepVGG, MCNet, CLDNN, and LSTM.

## 5 Experiment results and discussion

### 5.1 Model performance with different numbers of filters

**Fig 3:**
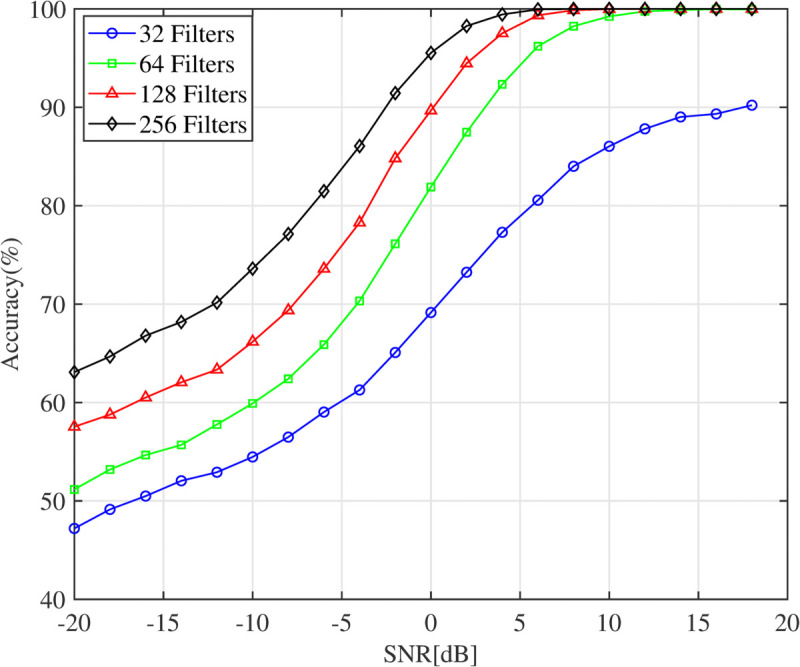
Accuracy model with filter difference.

In the first experiment, the AMC accuracy of the DFENet model is analyzed under the impact of different numbers of filters in the convolutional layers. The evaluation is conducted using the HisarMod2019 dataset. Specifically, the performance of the model is examined across varying SNR levels, with the number of filters in the convolutional layers adjusted to 32, 64, 128, and 256, as shown in Fig 3. The numerical results show a clear upward trend in classification accuracy as the number of filters increases, indicating that more filters enable the model to extract more informative features from the input data. The accuracy improved by approximately 6% with the increase in the number of filters, highlighting the model’s enhanced ability to differentiate between modulation schemes, especially in low SNR conditions. However, this improvement comes with a trade-off between AMC accuracy and computational complexity. Increasing the number of filters leads to a larger model and, consequently, greater computational complexity. It is also worth noting that when the number of convolutional filters reaches 256, the improvement in accuracy begins to approach saturation, with only a marginal gain of around 4%. This saturation suggests that further increases in the number of filters yield diminishing returns as the network approaches its maximum feature extraction capacity without significant improvements in classification performance. The effectiveness of the proposed CNN architecture lies in its ability to leverage larger convolutional filters to capture a wider range of signal characteristics. However, achieving the right balance between accuracy gains and computational efficiency is crucial when selecting the optimal number of filters. As a result of this experiment, choosing 128 filters provides the best trade-off between AMC accuracy and computational complexity.

### 5.2 Model performance with different signal lengths

**Fig 4:**
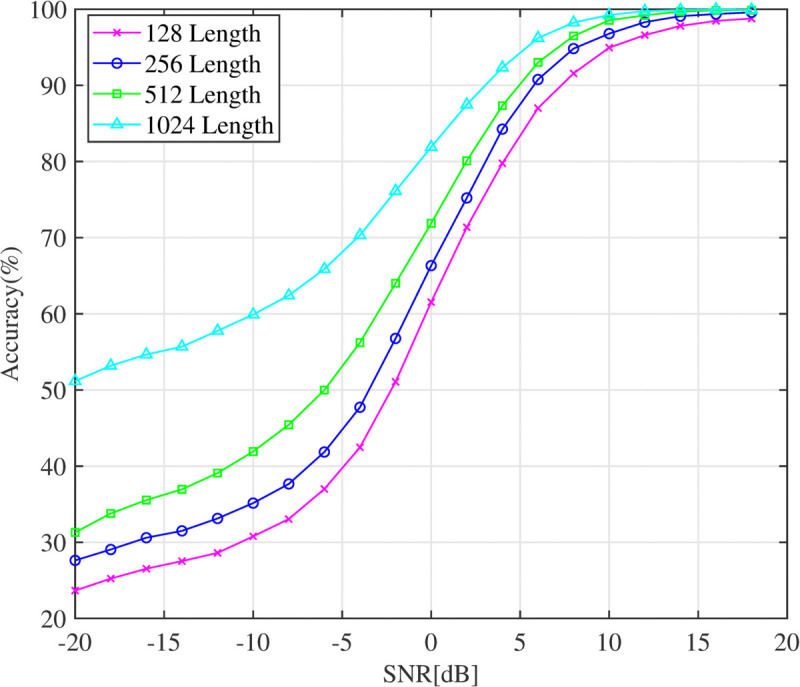
Classification accuracy with different values of signal length.

The length of the signal significantly impacts AMC performance in terms of accuracy and computational complexity. Therefore, the second experiment examines how varying signal lengths affects classification accuracy. Specifically, the DFENet model is evaluated at different SNR levels using signal lengths of 128, 256, 512, and 1024. The results illustrated in Fig 4 show that signal length plays a crucial role in classification accuracy, with longer signals consistently yielding better AMC performance. Longer signals provide more comprehensive and representative information about the underlying modulation schemes, allowing the model to differentiate between them more effectively. In contrast, shorter signals, such as those with a length of 128, limit the model ability to extract sufficient features, resulting in lower classification accuracy. In contrast, signals with a length of 1024 provide more detailed temporal patterns, enhancing the model’s ability to recognize complex modulations, even in noisy environments. However, while longer signal lengths improve classification results, they also increase computational load and processing time. In general, this experiment demonstrates that signal length is a critical parameter to optimize the performance of the DFENet model, with longer signals that prove advantageous for accurate modulation classification.

### 5.3 Model performance with different signal modulations

**Fig 5:**
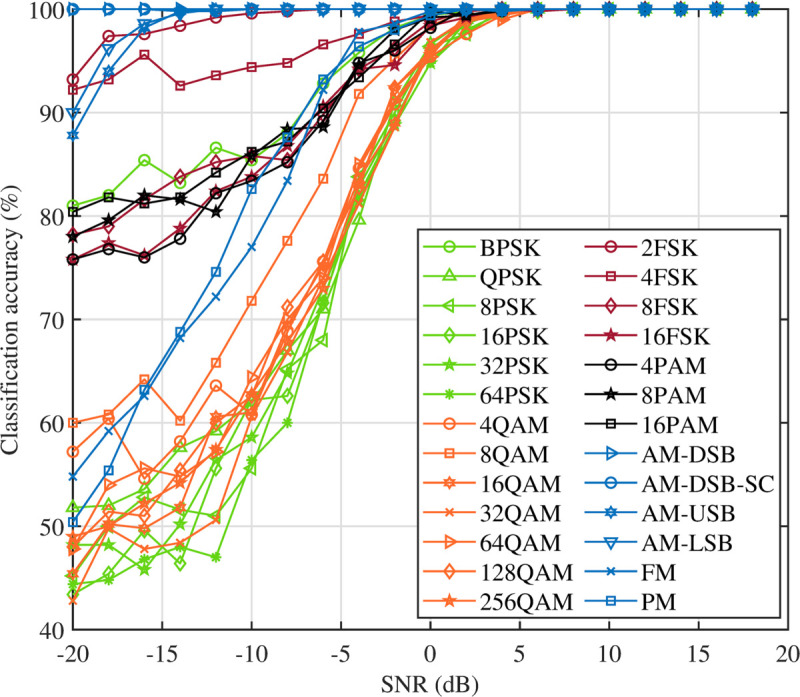
Accuracy performance of DFENet across 26 modulation types under varying SNR conditions.

In the third experiment, the AMC accuracy of the DFENet model is performed across 26 different modulation schemes. The results in Fig 5 indicate that modulation schemes with lower information rates, such as AM and FM, exhibit high classification accuracy under low SNR conditions. For example, AM achieves an accuracy rate 100% at –10 dB SNR, while FM and PM reach an accuracy of approximately 76%. This high performance can be attributed to the simpler nature of these modulation schemes, which enhances their robustness against noise.

In contrast, the DFENet model demonstrates effective recognition of the FSK and PAM signals, achieving accuracy rates of 87% and 83%, respectively, at –10 dB SNR. However, the AMC accuracy for the PSK and QAM signals decreases as the modulation order increases. This decrease is due to the increased vulnerability of higher-order modulation schemes to channel impairments and noise. Specifically, at –10 dB SNR, the accuracy rates for the PSK and QAM modulations decrease significantly to 54% and 61%, respectively. At a lower noise level of +0 dB, these accuracy rates improve markedly, reaching 94% for PSK and 96% for QAM.

These results highlight the effectiveness of the DFENet model in classifying lower-order modulation schemes under challenging conditions, while also underscoring the difficulty in accurately classifying higher-order modulation schemes, which are more susceptible to noise and channel distortions.

**Fig 6:**
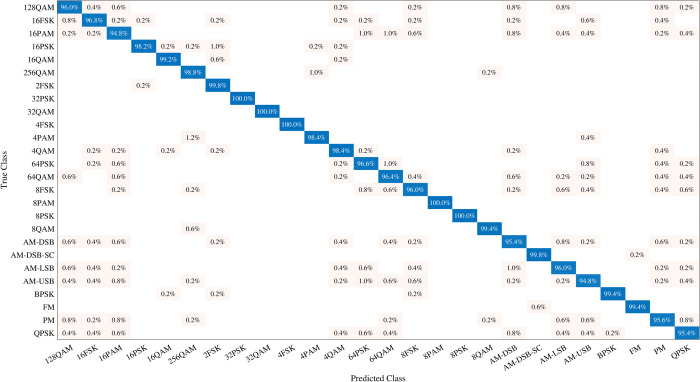
Confusion matrix of 26-modulation classification at +0 dB SNR.

**Fig 7:**
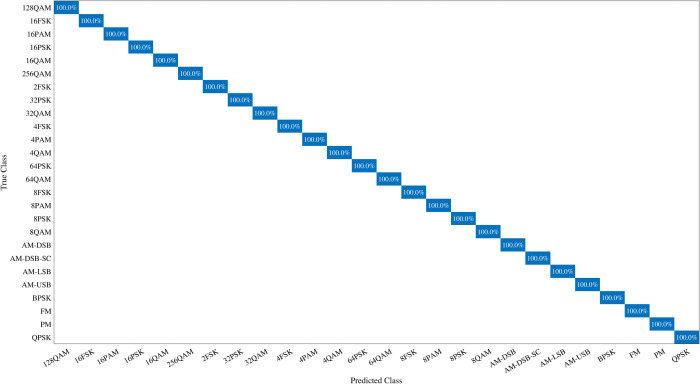
Confusion matrix of 26-modulation classification at +10 dB SNR.

The detailed analysis of confusion matrices for the 26 modulation classifications at +0 dB and +10 dB, as illustrated in Figs 6 and 7, provides critical insight into misclassification patterns among various modulation types. At +0 dB, modulation schemes such as PAM, FSK, and AM demonstrate minimal misclassifications and higher accuracy. As shown in the confusion matrix (Fig 6), the most notable errors occur between higher-order quadrature amplitude modulation (QAM) schemes, particularly between 64QAM and 16QAM. This confusion arises because under low-SNR conditions, the constellation points of 64QAM collapse toward each other and partially overlap with the constellation regions of lower-order QAM, making their statistical features difficult to separate. Such misclassification may lead to significant performance degradation in practical communication systems, since incorrect identification of a higher-order modulation as a lower-order scheme can directly affect demodulation, symbol decoding, and throughput estimation. Conversely, QAM and PSK modulation groups exhibit lower accuracy, with specific accuracies of 96% for 128QAM, 95.4% for QPSK, and 96.6% for 64PSK. Notably, the confusion matrix at +10 dB shows that the model achieves an impressive accuracy of 100% for most modulation types under lower noise conditions. This indicates that while the model performs exceptionally well with high accuracy at lower noise levels, certain modulation types such as QAM and PSK exhibit a slight decrease in accuracy as the complexity of modulation increases.

### 5.4 Feature space visualization via t-SNE

To gain insights into the feature separability and representational power of DFENet, the paper applies t-distributed Stochastic Neighbor Embedding (t-SNE) to the feature vectors extracted at two representative stages of the network: immediately after the first convolutional layer and at the output of the final fully connected layer.

**Fig 8:**
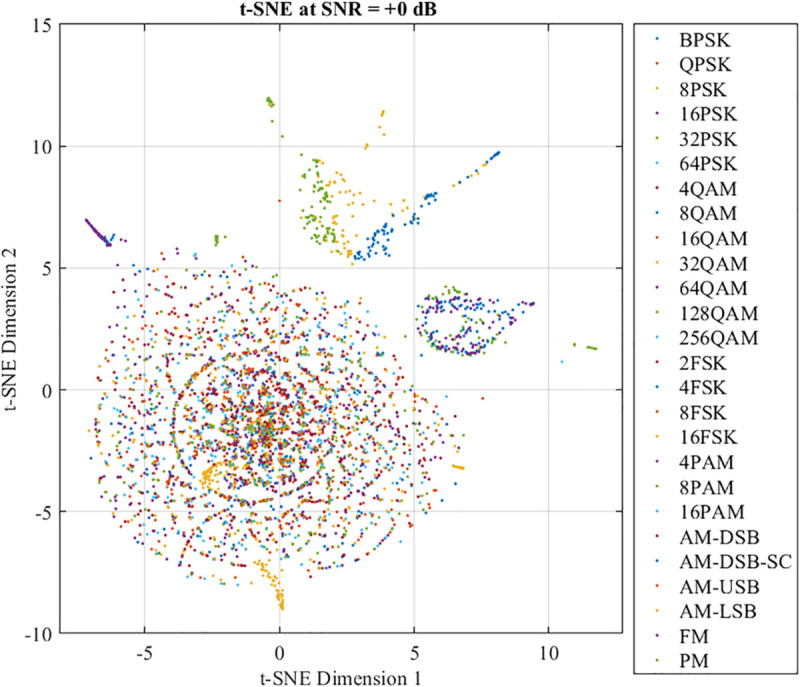
Output of the first convolutional layer at SNR = +0 dB.

Fig 8 illustrates the t-SNE projection of the feature representations extracted from the first convolutional layer at SNR = +0 dB. Although all 26 modulation types are present, the clusters are highly overlapping and lack clear separation boundaries. This indicates that low-level convolutional features primarily capture generic signal variations without class-specific discriminative power.

**Fig 9:**
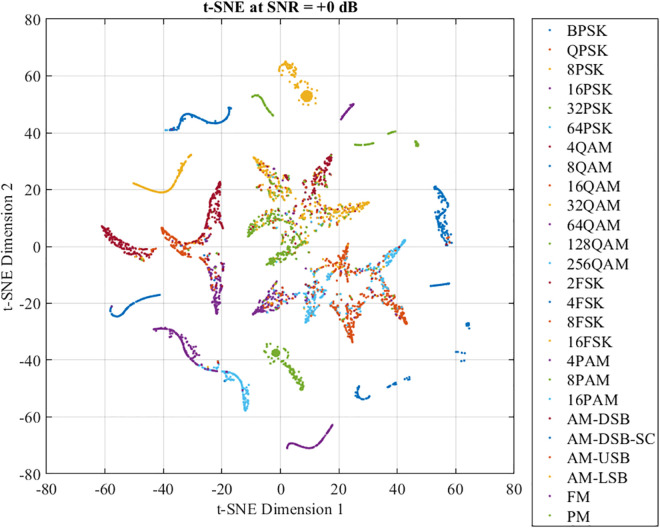
Output of the final fully connected layer at SNR = +0 dB.

In contrast, Fig 9 shows the t-SNE visualization at SNR = 0 dB. Despite the presence of severe noise, the network succeeds in forming partially compact clusters for many modulation types such as BPSK, QPSK, 2FSK, and FM, demonstrating its ability to learn low-level signal structures efficiently. However, significant overlaps are still observed among several modulation groups, particularly:

- Higher-order QAM (such as 64QAM, 128QAM, 256QAM) exhibit substantial cluster mixing in the t-SNE space. At low and medium SNR levels, the feature cluster corresponding to 64-QAM appears significantly less compact and exhibits substantial overlap with neighboring QAM clusters. This overlap indicates reduced separability in the latent feature space, caused by the very small Euclidean distance between constellation points in high-order QAM. Consequently, noise perturbs the amplitude and phase of 64-QAM symbols in a manner similar to other QAM schemes, preventing the network from forming a well-isolated representation. This lack of feature separability explains both the higher misclassification rate observed in the confusion matrix and the resulting degradation in effective throughput for 64-QAM.
- PAM types (such as 8PAM, 16PAM) are difficult to distinguish because of the linear amplitude.
- PSK schemes with increasing order (such as 16PSK, 32PSK) exhibit decreasing angular separation, resulting in interlayer mixing.
- AM-based modulations (such as AM-DSB, AM-USB, AM-LSB) have similar signal spectra.

These results indicate that, although DFENet begins to extract modulation features, full separability remains limited in high-noise regimes.

**Fig 10:**
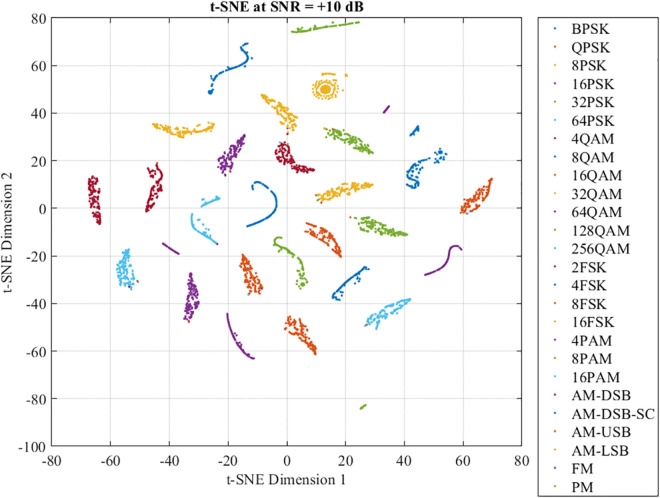
Output of the final fully connected layer at SNR = +10 dB.

Furthermore, Fig 10 demonstrates the t-SNE plot at the final FC layer with SNR = +10 dB. Here, a notable improvement in feature separability is observed. The results clearly distinguish between most modulation types, with feature clusters becoming more isolated and compact. Even previously confused modulation types, such as 64QAM and 16PAM, are separated. This significant improvement confirms that the hierarchical deep learning architecture of DFENet successfully transforms ambiguous low-level features into highly discriminative representations when signal quality is better. Overall, these t-SNE visualizations provide strong qualitative evidence that DFENet learns meaningful and structured representations, which evolve from noisy and overlapping to clean and separable as features propagate through the network and as channel conditions improve.

### 5.5 The effect of model structure

To validate the contribution of each architectural component, we conducted model component ablation experiments on the HisarMod2019 dataset. The DFENet model is evaluated under the following configurations:

Table 3 summarizes the ablation results of DFENet on the HisarMod2019 dataset, highlighting the impact of each architectural component on the accuracy of AMC. The full DFENet model, which incorporates both the multi-branch DFE blocks and the FEN refinement blocks, achieves the highest average accuracy of 80.8%. This demonstrates the effectiveness of combining multiscale feature extraction with deep feature enhancement for robust modulation classification.

**Table 3:** Ablation study on the HisarMod2019 dataset.

Model	Block		Multi-scale Filters	Avg. Accuracy (%)
	DFE	FEN		
DFENet-full	✓	✓	✓	**80.8**
No-FEN	✓	□	✓	70.31
No-DFE	□	✓	✓	70.59
No-multiscale	✓	✓	3×3 only	71.25
ShallowNet	□	□	□	14.95

When the FEN block is removed (No-FEN), the accuracy drops to 70.31%, confirming that batch normalization-based refinement plays a critical role in suppressing noise and stabilizing feature representations. Similarly, eliminating the DFE block results in 70.59%, confirming that multiscale feature extraction is essential for capturing temporal dependencies in IQ signals. Moreover, replacing the multiscale filters with a single 3×3 kernel (No-multiscale) reduces performance to 71.25%, indicating that diverse kernel sizes (1×1 to 7×7) significantly improve temporal context modeling. This confirms the benefits of extracting specific signals across different regulations.

Finally, the ShallowNet configuration performs the worst without any DFE, FEN, or multiscale structure, with only 14.95% accuracy. This baseline confirms that deeper structured feature hierarchies are essential for extracting discriminative modulation patterns. This result shows that shallow neural networks without fully dedicated processing blocks are inadequate to solve this problem. The nearly 66% performance improvement of DFENet over ShallowNet demonstrates the superior value and feature extraction capability of the multi-component architecture of our proposed model.

The results demonstrate that the ablation table effectively captures both the individual and aggregate contributions of each architectural component in DFENet. Furthermore, combining DFE and FEN blocks is the key to achieve significant performance gains in classification accuracy, especially under low SNR.

### 5.6 Model performance with different numbers of DFE blocks

**Fig 11:**
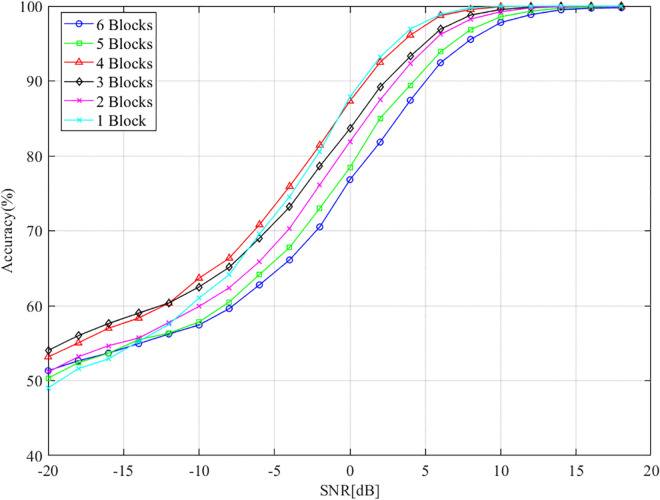
Classification accuracy with different numbers of DFE blocks.

In this experiment, we investigate the impact of varying the number of DFE blocks on the performance of the DFENet model, while keeping the number of convolutional filters fixed at 64. The evaluation provides insight into how structural modifications affect the accuracy of classification. The results in Fig 11 show that the benefit of adding more DFE blocks is most evident in the low-SNR regime (≤−5 dB), where accuracy improves consistently as the number of blocks increases from one to three. Specifically, adding an additional block results in an accuracy gain of approximately 2% in this range. At higher SNR values, however, the performance gap among different configurations becomes less pronounced. This indicates that the advantage of deeper feature extraction is particularly critical under noisy conditions, whereas in high-SNR scenarios, the signal patterns are already clear and discriminative, so further feature refinement provides limited benefits.

However, this trend does not continue indefinitely. When the number of DFE blocks is further increased from 4 to 6, the model’s accuracy begins to decline. In particular, the accuracy decreases by about 1% when the model is expanded from three to six blocks. This behavior suggests that while increasing the number of DFE blocks initially enhances the model’s ability to extract and utilize features, there is a diminishing return after a certain point. Overly complex models with excessive blocks may suffer from issues such as overfitting or increased computational overhead, which could counteract the benefits of additional feature extraction.

### 5.7 Evaluation metrics: Precision, Recall, and F1-score

**Fig 12:**
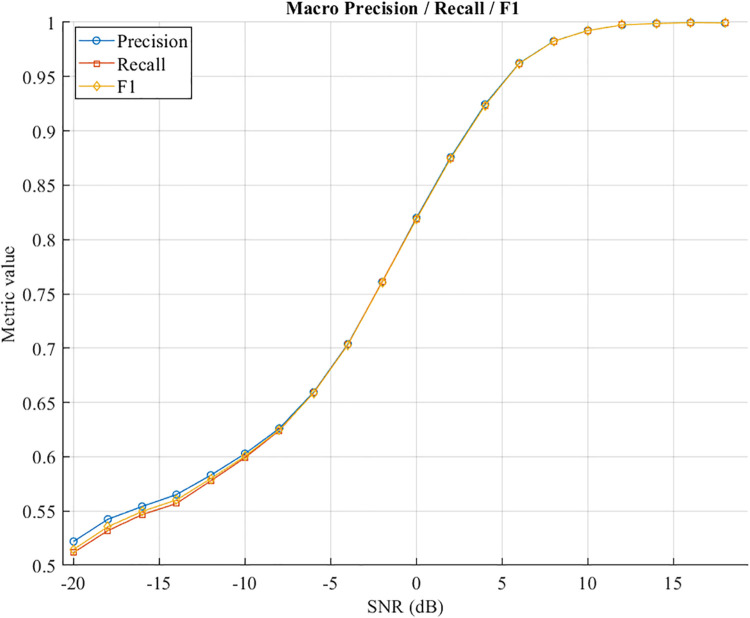
Macro averaged Precision, Recall, and F1-score of DFENet.

To complement overall accuracy and provide a more comprehensive evaluation of DFENet’s classification performance, we consider three key metrics: Precision, Recal, and F1-score, as shown in Fig 12. Let *TP*, *FP*, and *FN* denote the number of true positives, false positives, and false negatives, respectively. In the context of multiclass modulation classification, we define:

$$\text{Precision}=\frac{TP}{TP+FP}$$ (17)

$$\text{Recall}=\frac{TP}{TP+FN}$$ (18)

$$\text{F1-score}=2\cdot \frac{\text{Precision}\cdot \text{Recall}}{\text{Precision}+\text{Recall}}$$ (19)

Since modulation types are not uniformly distributed, we adopt macro-averaging to ensure equal treatment across all classes:

$$\text{Macro-F1}=\frac{1}{C}\sum_{i=1}^{C}{\text{F1}}_{i}$$ (20)

These metrics quantify different aspects of classification:

- Precision reflects how reliable a positive prediction is.
- Recall indicates how many actual instances are correctly captured.
- F1-score balances both and is particularly meaningful in AMC tasks where confusions often occur among high-order modulations.

At low SNRs, all three metrics improve consistently as SNR increases. At very low SNRs (e.g., −20 dB to 0 dB), DFENet maintains reasonable performance, with F1-score rising from 0.52 to around 0.80. This indicates that even under severe noise, the model can still capture meaningful patterns in the signal.

From 0 dB to +10 dB, the accuracy increases rapidly, and after +10 dB, it approaches 0.99, reflecting near-perfect classification of the modulated signal. The gap between precision and recall remains minimum at all SNR levels, indicating that the model does not favor any particular class and balances false positives and false negatives as well.

The gap between Precision and Recall remains minimal across all SNRs, indicating a balanced prediction behavior without significant bias toward false positives or false negatives. This suggests that DFENet not only correctly identifies modulation types. These results demonstrate that DFENet offers robust generalization across a wide range of signal conditions, and that its high F1 performance confirms both sensitivity and specificity in challenging wireless environments.

### 5.8 Comparison between DFEnet with other existing models

In the final experiment, we evaluated the DFE model using the HisarMod2019 and RadioML2018 datasets to assess its performance under diverse conditions and validate its generalization against contemporary models.

**Fig 13:**
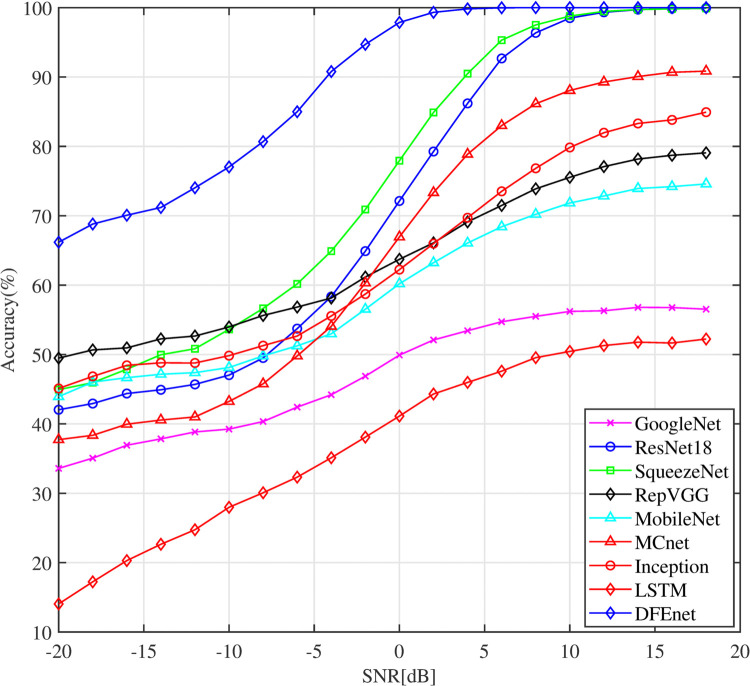
Comparison of the classification accuracy of different single CNN models for HisarMod2019 dataset.

We compared the AMC accuracy of the DFE model with existing models using the HisarMod2019 dataset, as illustrated in Fig 13. The LSTM model exhibited the lowest accuracy, achieving a maximum of only 52% at +18 dB SNR, due to its limited feature extraction capabilities. In contrast, GoogleNet demonstrated superior performance, benefiting from its multilayer convolutional (Conv) structure, which improves its learning capacity. Models such as MobileNet, RepVGG, and Inception also showed improved performance, with accuracy increases of approximately 20%, 27%, and 32% respectively, compared to LSTM, across SNRs ranging from –20 dB to +18 dB. Furthermore, models such as SqueezeNet, which uses multiple Conv layers with increasing filter sizes, ResNet, with residual connection blocks, and MCNet, with combined parallel residual blocks, achieved higher accuracies in the range of +2 dB to +18 dB. Notably, the DFE model achieved the highest accuracy, averaging 85.2%. It excelled particularly at low SNRs, with accuracy exceeding 60% at –20 dB and exceeding 90% at –2 dB. This exceptional performance of the IVD model is attributed to its effective feature extraction, supported by extended block sizes and various filter sizes, which results in superior classification accuracy compared to current models.

DFENet exhibits notable efficiency and performance compared to other models, as shown in Table 4. With a streamlined architecture, DFENet variants, such as DFENet-1block (1.3 million parameters) and DFENet-4block (2 million parameters), are significantly more compact compared to models like RepVGG and Inception, which have parameter counts of 24.8 million and 21.8 million, respectively. This compactness contributes to its efficient performance.

**Table 4:** Complexity and computational time of the models.

Model	Parameters(m)	Time (ms)
GoogleNet	5.9	1.361
ResNet18	11.1	1.218
SqueezeNet	2.4	1.186
RepVGG	24.8	3.189
MobileNet	2	2.290
MCnet	10.3	1.346
Inception	21.8	1.416
LSTM	1.1	0.9725
DFEnet-1block	1.3	1.338
DFEnet-2block	1.4	1.422
DFEnet-3block	1.7	1.485
DFEnet-4block	2	2.132

In terms of computational time, DFENet remains competitive. For example, DFENet 1block operates at 1.338 ms, which is on par with or faster than models such as SqueezeNet (1.186 ms) and ResNet18 (1.218 ms). Even as the number of blocks increases, as seen with DFENet-4block (2.132 ms), it maintains efficiency compared to other models such as MobileNet (2.290 ms) and RepVGG (3.189 ms). The scalability of DFENet is another strength. The block-based architecture allows for proportional increases in both parameters and computational time as more blocks are added. Despite this, DFENet effectively manages these increases, offering a balanced solution that adapts well to various application scenarios while maintaining high efficiency.

The efficient use of parameters by DFENet and its competitive computational speed highlight its strengths. Its design offers a balanced approach that integrates high performance with manageable computational demands, making it a suitable choice for applications that require both efficiency and speed.

A comprehensive evaluation of a deep learning model requires not only an assessment of its accuracy but also an analysis of its computational demands and practicality for real-world deployment. This section addresses the training complexity, memory requirements, and inference speed of the proposed DFENet model to demonstrate its feasibility.

The training and simulation experiments were conducted on a system equipped with a 3.70 GHz CPU, 2x16GB RAM, and a single NVIDIA GeForce RTX 3060 Ti GPU. For the HisarMod2019.1 dataset, models were trained for 20 epochs, while the RadioML2018.01A dataset required up to 90 epochs. The optimization was performed using SGDM (Stochastic Gradient Descent with Momentum) with a fixed learning rate of 0.001. The use of a consumer-grade GPU highlights that training the DFENet model is accessible and does not necessitate specialized, high-cost data-center-level hardware, confirming its efficient training profile. As detailed in Table 4, the complexity of the DFENet architecture is a key advantage. Our proposed DFENet-4block model consists of only 2.0 million parameters. This is a substantial reduction compared to other high-performing models like RepVGG (24.8 million) and Inception (21.8 million). This compact model size directly translates to a smaller memory footprint, which is a critical advantage for deployment on resource-constrained devices, such as those found in IoT networks and edge computing environments.

Beyond memory needs, the model’s inference speed is a crucial metric for practical applications. As shown in the clarified Table 4, the DFENet-4block model achieves a fast inference time of 2.132 ms per sample. This performance is highly competitive and validates the model’s suitability for time-sensitive tasks. The combination of a low parameter count and high inference speed confirms that DFENet provides an excellent balance between high classification accuracy and computational efficiency, making it a practical and robust solution for real-world AMC tasks.

**Fig 14:**
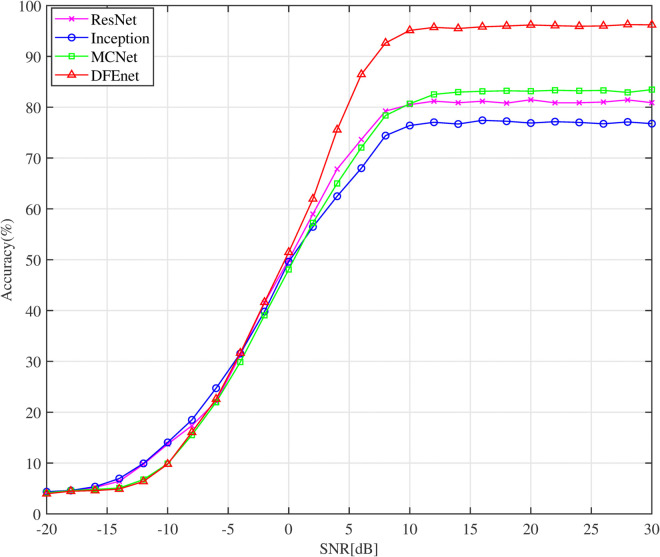
Comparison of the classification accuracy of different single CNN models for RadioMl2018 dataset.

We further evaluated the DFE model against three other models, including MCNet, Inception, and ResNet, using the RadioML2018 dataset, as shown in Fig 14. The Inception model demonstrated the lowest classification accuracy, reaching a maximum of only 73% at SNR = +18 dB. This limited performance is likely due to its suboptimal feature extraction capabilities. In contrast, the ResNet and MCNet models achieved higher precision levels due to their advanced residual connection structures, reaching accuracies of 81% and 83%, respectively, at SNR = +18 dB. These structures enhance the models’ ability to learn and stabilize feature extraction. Notably, the DFE model outperforms all tested models, achieving over 93% accuracy at SNR levels above +6 dB. The DFE model also demonstrated significant robustness at low SNR, with an AMC accuracy exceeding 60% at –20 dB and above 90% at –2 dB. This substantial improvement underscores the DFE model’s superior capability in handling noisy conditions and its effective feature extraction, making it different from existing models.

## 6 Conclusion

In this study, we introduced a state-of-the-art deep learning approach to improve automatic modulation classification under challenging channel conditions. The proposed DFENet architecture employs multi-branch diverse feature extraction blocks with parallel multi-scale filters, enabling more effective learning of discriminative signal representations. Extensive evaluations on two widely used public benchmarks, namely HisarMod2019 and RadioML2018, demonstrate that DFENet consistently outperforms existing methods in terms of classification accuracy while maintaining competitive computational efficiency.

Despite these encouraging results, the current evaluation is limited to simulation-based datasets. In future work, we plan to extend this study by validating DFENet on newly collected datasets obtained using software-defined radio (SDR) platforms and over-the-air (OTA) measurement testbeds. These datasets will enable quantitative analysis of the performance gap between simulation and real-world wireless channels, including practical impairments such as phase noise, carrier frequency offset, multipath fading, and hardware non-idealities. Such validation is essential to demonstrate the robustness and deployment potential of DFENet in real wireless communication systems. Furthermore, future research will explore encoder–decoder-based adversarial denoising frameworks to further enhance classification robustness in low-SNR environments.
